# Cold-Induced Thermogenesis and Inflammation-Associated Cold-Seeking Behavior Are Represented by Different Dorsomedial Hypothalamic Sites: A Three-Dimensional Functional Topography Study in Conscious Rats

**DOI:** 10.1523/JNEUROSCI.0100-17.2017

**Published:** 2017-07-19

**Authors:** Samuel P. Wanner, M. Camila Almeida, Yury P. Shimansky, Daniela L. Oliveira, Justin R. Eales, Cândido C. Coimbra, Andrej A. Romanovsky

**Affiliations:** ^1^Systemic Inflammation Laboratory (FeverLab), Trauma Research, St. Joseph's Hospital and Medical Center, Phoenix, Arizona 85013,; ^2^Exercise Physiology Laboratory, School of Physical Education, Physiotherapy and Occupational Therapy, Federal University of Minas Gerais, Belo Horizonte, MG 31270-901, Brazil,; ^3^Kinesiology Program, Arizona State University, Phoenix, Arizona 85004, and; ^4^Endocrinology and Metabolism Laboratory, Institute of Biological Sciences, Federal University of Minas Gerais, Belo Horizonte, MG 31270-901, Brazil

**Keywords:** body temperature, cold defense, dorsomedial hypothalamus, hypothermia, lipopolysaccharide, systemic inflammation, thermoregulation, thermoregulatory behavior

## Abstract

In the past, we showed that large electrolytic lesions of the dorsomedial hypothalamus (DMH) promoted hypothermia in cold-exposed restrained rats, but attenuated hypothermia in rats challenged with a high dose of bacterial lipopolysaccharide (LPS) in a thermogradient apparatus. The goal of this study was to identify the thermoeffector mechanisms and DMH representation of the two phenomena and thus to understand how the same lesion could produce two opposite effects on body temperature. We found that the permissive effect of large electrolytic DMH lesions on cold-induced hypothermia was due to suppressed thermogenesis. DMH-lesioned rats also could not develop fever autonomically: they did not increase thermogenesis in response to a low, pyrogenic dose of LPS (10 μg/kg, i.v.). In contrast, changes in thermogenesis were uninvolved in the attenuation of the hypothermic response to a high, shock-inducing dose of LPS (5000 μg/kg, i.v.); this attenuation was due to a blockade of cold-seeking behavior. To compile DMH maps for the autonomic cold defense and for the cold-seeking response to LPS, we studied rats with small thermal lesions in different parts of the DMH. Cold thermogenesis had the highest representation in the dorsal hypothalamic area. Cold seeking was represented by a site at the ventral border of the dorsomedial nucleus. Because LPS causes both fever and hypothermia, we originally thought that the DMH contained a single thermoregulatory site that worked as a fever–hypothermia switch. Instead, we have found two separate sites: one that drives thermogenesis and the other, previously unknown, that drives inflammation-associated cold seeking.

**SIGNIFICANCE STATEMENT** Cold-seeking behavior is a life-saving response that occurs in severe systemic inflammation. We studied this behavior in rats with lesions in the dorsomedial hypothalamus (DMH) challenged with a shock-inducing dose of bacterial endotoxin. We built functional maps of the DMH and found the strongest representation of cold-seeking behavior at the ventral border of the dorsomedial nucleus. We also built maps for cold-induced thermogenesis in unanesthetized rats and found the dorsal hypothalamic area to be its main representation site. Our work identifies the neural substrate of cold-seeking behavior in systemic inflammation and expands the functional topography of the DMH, a structure that modulates autonomic, endocrine, and behavioral responses and is a potential therapeutic target in anxiety and panic disorders.

## Introduction

The tight control of deep body temperature (*T*_b_) observed in homeothermic animals is achieved by the multisensor, multieffector thermoregulation system, which functions as a federation of relatively independent thermoeffector loops (Romanovsky, 2007b; Werner, 2010). The effectors include autonomic (physiological) and behavioral and each of them is controlled by a unique combination of signals from peripheral and central thermosensors (for review, see Romanovsky, 2014) through an independent neural pathway (Roberts, 1988; McAllen et al., 2010). The neural pathways for autonomic thermoeffectors in rodents are well described (Morrison, 2016), but little is known about the pathways controlling thermoregulatory behaviors.

Systemic inflammation, the main cause of death in hospitalized patients, changes the regulation of *T*_b_ in such a way that either fever or hypothermia occurs (Romanovsky et al., 2005; Rudaya et al., 2005; Steiner and Romanovsky, 2007). In rodent models, aseptic systemic inflammation is often induced by intravenous administration of bacterial lipopolysaccharide (LPS, an endotoxin), which causes fever, hypothermia, or a combination of the two depending on LPS dose and ambient temperature (*T*_a_). Lower doses of LPS, especially when administered in a thermoneutral or warm environment, typically cause fever. Higher, shock-inducing doses of LPS, especially when administered in a subneutral (cool) environment, cause hypothermia. The two main effector mechanisms for LPS hypothermia are inhibition of thermogenesis on the autonomic side (Romanovsky et al., 1996b) and active cold seeking on the behavioral side (Romanovsky et al., 1996b; Almeida et al., 2006a).

Whereas fever is associated with motor agitation, arterial hypertension, arousal, and hyperalgesia, the hypothermic response is associated with motor depression, hypotension, sleepiness, and analgesia (Romanovsky et al., 1996a; Romanovsky and Székely, 1998). These two syndromes, which we termed the early and the late sickness syndrome, respectively, correspond to the light and severe forms of systemic inflammation. They also represent two types of adaptation to inflammation or infection: fight/flight (energy expenditure) and depression/withdrawal (energy conservation). Hypothermia is a central symptom of the late sickness syndrome. When rats are allowed to seek their preferred *T*_a_ (as in a thermogradient apparatus) to decrease their *T*_b_ (Romanovsky et al., 1997) or when they are cooled by being placed at a lower *T*_a_ (Liu et al., 2012), their survival rate in severe systemic inflammation increases drastically.

Even though the hypothermic response in systemic inflammation can be a lifesaver, it is not well studied. In particular, its neural mechanisms are unknown except that LPS-induced cold-seeking behavior in rats has been found to be mediated by neuronal bodies located in the dorsomedial hypothalamus (DMH) and neuronal fibers passing through the paraventricular hypothalamus (PVH) (Almeida et al., 2006b). Interestingly, the DMH and PVH are the only two diencephalic areas that show a stronger expression of c-Fos protein, a marker of neuronal activation, during LPS hypothermia than during LPS fever (Wanner et al., 2013).

There is a contradiction, however. Almeida et al. (2006b) reported that the same DMH lesion both promoted hypothermia (in cold-exposed restrained rats) and blocked it (in rats challenged with LPS in a thermogradient apparatus). Knowing that DMH neurons in and around the dorsal hypothalamic area (DA) mediate cold defenses, especially nonshivering thermogenesis in brown adipose tissue (BAT) and shivering (DiMicco and Zaretsky, 2007; Morrison, 2016), we thought that a single thermoregulation-related site (DA) was responsible for both phenomena reported by Almeida et al. (2006b) and possibly determined the direction of the thermoregulatory response (fever or hypothermia) in inflammation. We thought that, when the DA was lesioned, the animal could neither increase thermogenesis in response to cold nor inhibit thermogenesis in response to a high dose of LPS. Because the cold-seeking behavior is often coupled with metabolic inhibition (Romanovsky, 2004), the same DA lesion could also result in suppressed cold seeking. The present study rejects this explanation. It shows that DA neurons that drive thermogenesis are not involved in LPS-induced cold seeking and that cold-seeking behavior in systemic inflammation depends on a different, previously unknown, neuronal group.

## Materials and Methods

### 

#### Animals

Experiments were performed in 225 adult male Wistar rats (Harlan Laboratories). At the time of electrolytic, thermal, or chemical lesioning of the DMH, the rats had a body mass of 280–320 g. Rats were housed in standard “shoe box” cages placed in a rack equipped with a Smart Bio-Pack ventilation system and Thermo-Pak temperature control system (Allentown Caging Equipment). The incoming air temperature was maintained at 28°C. The room was on a 12:12 h light-dark cycle (lights on at 7:00 A.M.). The rats had *ad libitum* access to tap water and standard rat chow (Teklad diet; Harlan Laboratories). All rats were extensively habituated to the experimental setups as follows. Each rat designated for an experiment in the thermocouple or respirometry setup (see “Experimental setups” section) was habituated to staying in a wire mesh confiner by going through 8 training sessions of increasing duration (from 30 min to 4 h). The confiner limited back-and-forth movement and prevented the rat from turning around. The same confiners were used later in the experiments. Each rat designated for an experiment in the thermogradient apparatus was acclimated to a channel of the apparatus by going through 8 habituation sessions of increasing duration (from 2 to 8 h); during long (8 h) habituation sessions, rats had *ad libitum* access to food and water. On the day before an experiment, rats were placed inside the apparatus at 6:00 P.M. and left there overnight to further acclimate to the experimental conditions. At the end of experiments, rats were killed by exsanguination under anesthesia with a ketamine–xylazine–acepromazine mixture (55.6, 5.5, and 1.1 mg/kg, respectively, i.p.). All protocols were approved by the St. Joseph's Hospital and Medical Center Animal Care and Use Committee.

#### Surgery and brain tissue lesioning

##### Anesthesia and perioperative care.

For all surgical and brain lesioning procedures, the rats were anesthetized with ketamine–xylazine–acepromazine (55.6, 5.5, and 1.1 mg/kg, respectively, i.p.) and received an antibiotic prophylactically (enrofloxacin, 1.2 mg/kg, s.c.). During surgery, each rat was kept on a Deltaphase isothermal pad (Braintree Scientific) to prevent hypothermia. After surgery, all rats were examined daily for signs of dehydration (which often occurred in rats with large bilateral electrolytic DMH lesions). If a rat lost >15% of its body mass over 24 h after brain lesioning or exhibited a slow recovery from the initial loss of body mass, it received isotonic saline (10 ml, s.c.).

##### Electrolytic brain tissue lesioning.

To produce a large bilateral electrolytic lesion of the DMH, a rat was anesthetized and the head skin over the frontal and parietal bones was shaved and scrubbed. The rat was fixed to a stereotaxic apparatus (David Kopf Instruments) with the incisor bar set at 3.3 mm ventral to interaural line. The skin was incised over the sagittal suture and the periosteum was detached from the bone and excised. The level of the incisors bar was adjusted to make sure that bregma and lambda were at the same level. A small hole was drilled on each side of the skull using an anteroposterior coordinate (AP) of −3.0 mm and a mediolateral coordinate (ML) of 0.5 mm (Paxinos and Watson, 2007). A stainless steel electrode (250 μm diameter; Frederic Haer) was inserted into the brain through the drilled hole to a dorsoventral coordinate (DV) of −9.0 mm. A second electrode (an “alligator” clip) was attached to the edge of the surgical wound on the head. To lesion the brain tissue, a precision lesioning instrument (Ugo Basile) was used. A constant anodal current (1 mA) was passed through the electrodes for 30 s. After the structure of interest was lesioned on one side, the electrode was removed and inserted contralaterally. After the second lesion was made, the electrode was removed and the scalp wound was sutured. Sham-lesioned rats were prepared similarly, but the tip of the electrode was placed at a DV coordinate of −7.0 mm (just above the mammillothalamic tract) and no current was passed.

##### Thermal brain tissue lesioning.

Small bilateral thermal lesions within the DMH were produced by the passage of an alternating (radiofrequency) current. A rat was prepared as for electrolytic lesioning; holes were drilled into the skull and a probe consisting of an electrode (to lesion tissue) and a thermocouple (to measure tissue temperature inside the lesion) was inserted into the brain. To place lesions in different areas, throughout the entire DMH, the coordinates used varied widely between rats: AP, from −2.28 mm rostrally to −3.60 mm caudally; ML, from 0.20 mm medially to 0.80 mm laterally; and DV from −7.60 mm dorsally to −9.20 mm ventrally. The electrode was connected to a generator RFG-4A (Radionics) and a current was rapidly (within a few seconds) increased from 0 to a value corresponding to the brain temperature of 70–75°C and kept at this level for 45 s. Thereafter, the probe was removed and inserted to the same coordinates contralaterally. Sham-lesioned rats were prepared similarly, but the tip of the probe was placed at a DV coordinate of −7.0 mm and no current was passed.

##### Chemical (excitotoxic) brain tissue lesioning.

Small bilateral chemical lesions within the DMH were produced by ibotenic acid. To prevent the cardiovascular side effects of the intrabrain administration of ibotenic acid, rats were pretreated with a ganglionic blocker (hexamethonium, 30 mg/kg, i.p.). For lesioning the targeted area within the DMH, a stainless steel injector needle [outer diameter (OD) 210 μm] was inserted to the following tip coordinates: AP = −3.48 mm; ML = 0.40 mm (or −0.40 mm); and DV = −9.00 mm. Ibotenic acid (10 ng/nl) in 0.01 m PBS, pH 7.4, was delivered (7.5 nl/min, 10 min) with the help of an infusion pump (model 220; KD Scientific) and the injector needle was left in place for an additional 5 min. Sham-lesioned rats were prepared the in same way, but received an infusion of PBS instead of ibotenic acid.

##### Jugular catheterization.

Ten days after brain tissue (or sham) lesioning, each rat was anesthetized for a second time. A 1 cm longitudinal incision was made on the ventral surface of the neck and the left jugular vein was exposed, freed from its surrounding connective tissue, and ligated. A silicone catheter (OD 0.9 mm) filled with heparinized pyrogen-free saline (10 U/ml) was passed into the superior vena cava through the jugular vein and secured in place with ligatures. The free end of the catheter was knotted, tunneled under the skin to the nape, and exteriorized. The surgical wound on the ventral surface of the neck was sutured. After jugular catheter implantation, the catheter was flushed with heparinized saline daily (0.3 ml; 10 U/ml).

##### Temperature data logger implantation.

Each rat assigned to an experiment in the thermogradient setup was implanted with a miniature temperature data logger (SubCue Dataloggers) to record abdominal temperature. Immediately after jugular catheterization (under the same anesthesia), a midline laparotomy was performed and a data logger was inserted in the peritoneal cavity and sutured to the lateral abdominal wall. The data logger had been programmed to acquire data every 5 min. The surgical wound was sutured in layers.

#### Experimental setups

##### Thermocouple thermometry setup.

A rat was placed in a confiner and equipped with a copper–constantan thermocouple (Omega Engineering) to measure deep (colonic) *T*_b_. The colonic thermocouple was inserted 10 cm beyond the anal sphincter and fixed to the base of the tail with a loop of adhesive tape. Six rats instrumented with colonic thermocouples were placed simultaneously in their confiners inside a climatic chamber (model 3940; Forma Scientific). Thermocouple wires were passed through a wall port outside the chamber and fed into a data logger (Cole-Parmer). Based on the method developed in our laboratory (Romanovsky et al., 2002), we determined in preliminary experiments that the thermoneutral zone for the rats used in this study in this setup was centered at ∼28°C, which is 1.8°C lower than in our earlier study (Romanovsky et al., 2002). The midpoints of the thermoneutral zones for adult male Wistar rats in all experimental setups used are shown in Table 1.

**Table 1.** Experiments performed, parameters recorded, and ambient temperatures used in each experimental setup

Setup	Experiment	Parameter(s) recorded	Ambient temperature(s) used	Midpoint of the thermo-neutral zone*^a^* (reference)
Thermocouple	Electrolytic (or sham) DMH lesion + cold exposure	Colonic *T*_b_	28°C (neutral) → 10°C (deep subneutral)	28°C (current study)
	Electrolytic (or sham) DMH lesion + heat exposure		26°C (light subneutral) → 32°C (supraneutral)	
	Thermal (or sham) DMH lesion + cold exposure		28°C (neutral) → 9°C (deep subneutral)	
	Chemical (or sham) DMH lesion + cold exposure		28°C (neutral) → 7°C (deep subneutral)	
Respirometry	Electrolytic (or sham) DMH lesion + LPS, 5000 μg/kg (or saline), i.v.	Colonic *T*_b_, tail *T*_sk_, HLI, VO_2_	21°C (subneutral)	26°C (Steiner et al., 2007)
	Electrolytic (or sham) DMH lesion + LPS, 10 μg/kg (or saline), i.v.		26°C (neutral)	
Thermogradient	Electrolytic (or sham) DMH lesion + LPS, 5000 μg/kg, i.v.	Abdominal *T*_b_, preferred *T*_a_	Freely selected from 15–30°C (subneutral to supraneutral)	24°C (Almeida et al., 2006a)
	Electrolytic (or sham) DMH lesion + LPS, 10 μg/kg, i.v.			
	Thermal (or sham) DMH lesion + LPS, 5000 μg/kg, i.v.			
	Chemical (or sham) DMH lesion + LPS, 5000 μg/kg, i.v.			

For each ambient temperature, its relation to the thermoneutral zone in the given setup is indicated.

*^a^*Please note that the same animal has different thermoneutral zones in different experimental setups because the heat exchange between the body and the environment depends, not only on the *T*_a_, but also on several other physical factors (Romanovsky et al., 2002; Romanovsky, 2007a).

##### Respirometry setup.

A rat was placed in a confiner and equipped with a colonic thermocouple (as in the thermocouple setup) and a tail-skin thermocouple. The latter was positioned on the lateral surface of the tail, at the boundary of the proximal and middle thirds and insulated from the environment with tape. Each rat had a preimplanted jugular catheter, which was connected to a PE-50 extension filled with LPS suspension (in saline) or saline. Each rat in its confiner was then placed inside a cylindrical Plexiglas metabolic chamber (Sable Systems). The catheter extension and thermocouple wires were passed through a port of the metabolic chamber. The port was sealed with paraffin and the metabolic chamber was ventilated continuously. The airflow was maintained at 600 ml/min (standard conditions for temperature and pressure) with the aid of a mass flow controller (Sierra Instruments). The air leaving each metabolic chamber was sampled automatically, dried, and passed through an oxygen analyzer (Sable Systems). Six metabolic chambers were placed inside a large climatic chamber, which allowed for the study of six rats simultaneously. The catheter extensions and thermocouple wires were then passed through a wall port of the climatic chamber. Each extension was connected to a syringe loaded onto an infusion pump (model 220; KD Scientific) and the thermocouples were plugged into the data logger. *T*_a_ values of 25.0–27.0°C (as measured inside the environmental chamber, but outside the metabolic chambers) are neutral for rats in this setup (Table 1).

##### Thermogradient setup.

The thermogradient apparatus used was described in detail previously (Almeida et al., 2006a). Briefly, this apparatus consists of six 200-cm-long aluminum channels with acrylic double-walled lids at the top. The raised floor of each channel is made of a stainless-steel grid and hosts an animal during experiments. At each end, all channels share a common aluminum wall, which separates the channels from a large tank. The tank at the “warm” end of the channels is filled with water heated by two electric heating units (PolyScience) to maintain air temperature inside the channels at this end at 30.0°C. The tank at the opposite (“cold”) end is perfused constantly with 30% ethylene glycol by an external circulation cooling/heating pump (model 9501; PolyScience) to maintain air temperature inside the channels at this end at 15.0°C. In this setting, all channels have a common, nearly linear longitudinal temperature gradient of 0.075°C/cm. In each channel, the rat's position is monitored with 56 infrared emitters (which send transversal infrared beams) evenly spaced (3.5 cm apart) on one long wall of the channel and the corresponding 56 receivers on the opposite wall. The thermoneutral zone for adult male Wistar rats in our apparatus is centered at ∼24°C (Table 1), which is a typical preferred *T*_a_ of well adapted rats in this setup (Almeida et al., 2006a).

#### Experiments

In the thermocouple setup, we studied the ability of restrained rats with electrolytic, thermal, or excitotoxic (ibotenic acid) lesions and of their corresponding sham-lesioned controls to defend their *T*_b_ against cold or heat by using autonomic (physiological) effectors only. To test autonomic defense against cold, we first kept the rats at a neutral *T*_a_ of ∼28°C for 90 min and then gradually lowered it to 7–10°C. In experiments in rats with electrolytic and excitotoxic lesions, the cooling was performed over 120 min. In experiments with thermal lesions, we wanted to increase the magnitude of the hypothermic response (to achieve a better resolution of the functional maps) by exposing rats to cold for a longer time, so the cooling was performed over 150 min. To study the autonomic defense against heat (these experiments were performed only in rats with electrolytic lesions of the DMH and in their sham-lesioned controls), we first kept the rats in a slightly subneutral environment (*T*_a_ of ∼26°C) for 90 min and then gradually (over 120 min) raised the *T*_a_ to ∼32°C. Colonic *T*_b_ was recorded continuously (Table 1).

In the respirometry setup, rats were subjected to two experiments designed to investigate whether the electrolytic DMH lesions impaired their ability to recruit autonomic thermoeffectors in the thermoregulatory responses to LPS: hypothermia and fever. Hypothermia was caused by a bolus injection of a high, shock-inducing dose of LPS (5000 μg/kg, i.v.) in a cold environment (*T*_a_ of 21°C). Fever was induced by administering a low, pyrogenic dose of LPS (10 μg/kg, i.v.) at thermoneutrality (*T*_a_ of 26°C). In control experiments, pyrogen-free saline was injected in DMH- or sham-lesioned rats at the corresponding *T*_a_. The rate of oxygen consumption (VO_2_), colonic *T*_b_, and tail-skin temperature (*T*_sk_) were recorded continuously (Table 1).

In the thermogradient setup, two experiments were conducted. First, we investigated whether electrolytic DMH lesions (compared with sham lesions) impaired the ability of rats to recruit the warmth-seeking behavior in response to the low, pyrogenic dose of LPS (10 μg/kg, i.v.), which typically causes polyphasic fever and warmth seeking (Almeida et al., 2006a). The controls received saline instead of LPS. Second, we investigated whether electrolytic, thermal, and chemical lesions within the DMH impaired the ability of rats to recruit the cold-seeking behavior in response to the high, shock-inducing dose of LPS (5000 μg/kg, i.v.). High doses of LPS typically cause hypothermia and cold seeking (Romanovsky et al., 1996b; Almeida et al., 2006a). Abdominal *T*_b_ and preferred *T*_a_ were recorded (Table 1).

#### Drugs and drug administration

A stock suspension of *E. coli* 0111:B4 LPS in pyrogen-free saline (10 mg/ml) was stored at 4°C. On the day of the experiment, the stock was diluted to a final concentration of 10 or 5000 μg/ml. In the experiments conducted in the thermogradient setup, LPS (10 μg/kg, i.v., to cause fever or 5000 μg/kg, i.v., to cause hypothermia) or saline (1 ml/kg, i.v.) was administered as a bolus through the jugular catheter after the rat was gently restrained by hand in its channel of the thermogradient apparatus. In the experiments conducted in the respirometry setup, LPS (same doses) or saline was bolus infused through a jugular catheter extension without disturbing the rat.

#### Histological verification

Rats were deeply anesthetized with ketamine–xylazine–acepromazine and perfused (15 ml/min) through the ascending aorta (right atrium cut) with 75 ml of saline, followed by 300 ml of 10% formalin. The brains were removed, postfixed in 10% formalin at 4°C overnight, and transferred to 30% sucrose in 0.01 m PBS, pH 7.4, thereafter. To ensure cryoprotection, the brains were kept in the sucrose solution at 4°C for 48 h and then frozen on dry ice and sectioned into 50 μm slices. Sections containing the structures of interest were collected, mounted on glass slides, and stained with cresyl violet (which stains Nissl bodies in the cytoplasm of neurons purple–blue). Multiple photomicrographs of the slides containing lesions were taken for all lesioned brains.

#### Measuring the size of a thermal lesion

The center of a thermal lesion was determined as an estimated center of mass of the lesion's image on the slide where the lesion was largest. For a round lesion, this was the geometric center. To determine the stereotaxic coordinates of the center, a photomicrograph of the section containing the lesion center was superimposed on the best-matched coronal schematic from the digitized Paxinos and Watson (2007) atlas using Microsoft PowerPoint. The anatomical landmarks used to find the best-matching image from the atlas included the mammillothalamic tract, fornix, dorsomedial hypothalamic nucleus (DM), especially its compact portion (DMC), third ventricle, and median eminence. The same schematic was used to determine the lesion diameter.

#### Data processing and analysis

##### Heat loss index (HLI).

The HLI was used as a measure of tail-skin vasomotor tone. It was calculated according to the following formula: HLI = (*T*_sk_ − *T*_a_)/(*T*_b_ − *T*_a_) (Romanovsky et al., 2002).

##### Thermogenesis.

As in the past (Garami et al., 2011; Almeida et al., 2012), VO_2_ was used as an index of thermogenesis. It was calculated by comparing the oxygen fraction in the air that exited the metabolic chamber occupied by the rat of interest (FO_2-rat_) to the oxygen fraction in the air that exited an empty chamber (FO_2-chamber_). The following formula was used: VO_2_ = [air flow × (FO_2-chamber_ − FO_2-rat_)]/(1 − [(1 − respiratory quotient) × FO_2-chamber_])/rat's body mass, where the respiratory quotient was considered to be 0.71. The equation term that includes the respiratory quotient accounts for the fact that carbon dioxide produced by the rat was not extracted from the air that was passed though the oxygen analyzer in our experimental setup.

##### Assessment of autonomic cold-defense impairment.

The relative decrease in deep (colonic) *T*_b_ was calculated as an index of autonomic cold-defense impairment for all thermally lesioned rats and used for computing representation maps of the DMH for the autonomic cold defense. First, the *T*_b_ change during the cold exposure was determined for each rat as the difference between the mean *T*_b_ for the last 10 min of and the last 10 min just before the cold exposure. The mean change in *T*_b_ of 10 sham-lesioned rats was also calculated. Then, for each DMH-lesioned rat, the relative decrease in *T*_b_ during the cold exposure was calculated as a difference between the mean change in *T*_b_ of 10 sham-lesioned rats and the individual change in *T*_b_ of the rat of interest. This number was used as a measure of the autonomic impairment caused by the lesion: a strong impairment (i.e., the rat could not defend its *T*_b_) corresponded to a high positive value, whereas no impairment corresponded to a near-zero value (i.e., the rat defended its *T*_b_ as strongly as the controls) or even a negative value (i.e., the rat defended its *T*_b_ against cold even better than the controls).

##### Assessment of impairment of LPS-induced cold-seeking behavior.

For all thermally lesioned rats, an index of impairment of the cold-seeking response to LPS was calculated as a relative increase in preferred *T*_a_. This index was used for computing representation maps of the inflammation-associated cold-seeking behavior in the DMH. For each rat, the lowest *T*_a_ that it selected in the thermogradient apparatus between 25 and 65 min after LPS administration was determined. This time interval corresponded to the period of the most pronounced cold-seeking behavior exhibited by rats in response to the same dose of LPS (Almeida et al., 2006a, 2006b). The mean lowest *T*_a_ of 10 sham-lesioned rats was also calculated. For each DMH-lesioned rat, the relative change in preferred *T*_a_ was then determined as the difference between the lowest *T*_a_ of this lesioned rat and the mean lowest *T*_a_ of 10 sham-lesioned rats. The resultant number showed whether the cold-seeking behavior of the lesioned rat in response to LPS was stronger (no impairment; negative values), weaker (positive values), or the same (0) compared with sham-lesioned rats.

##### Construction of functional representation maps.

Based on the functional impairment values obtained in 83 rats with bilateral thermal lesions and 10 rats with sham lesions, we computed DMH maps for the autonomic cold-defense and the LPS-induced cold-seeking behavior as follows. The lesions on the two sides of the brain received the same impairment value and, for mapping purposes, were treated as two lesions. The number of lesions used to compute the autonomic cold-defense maps was 166; the number of lesions used to compute the cold-seeking maps was 162. The mapped brain space extended from AP = −1.81 mm rostrally to AP = −4.49 mm caudally, from ML = 0.00 mm medially to ML = 1.37 mm laterally, and from DV = −6.86 mm dorsally to DV = −9.97 mm ventrally, thus covering a volume of 11.42 mm^3^. This space was conceptualized as a 3D grid with equally spaced nodes (0.01 mm apart along each of the AP, ML, and DV axes) and the nodes were treated as points within this space. Each thermal lesion was approximated with a ball within this space. The Paxinos and Watson (2007) coordinates of the center and the diameter of each ball were determined as described above (see “Measuring the size of thermal lesions” section). Each point within each ball was assigned a functional value equal to either the relative decrease in *T*_b_ during cold exposure (an index of the autonomic impairment) or the relative increase in the preferred ambient temperature after LPS administration (an index of the behavioral impairment), as obtained from experiments in the rat with that particular lesion. For each point in the mapped brain space, a subset of lesion balls containing this point was determined and, because each lesion had a functional value, this procedure defined the corresponding subset of functional values for this point. Next, from this subset of values, we computed a single functional value for the corresponding point of the mapped brain space. The mathematical formula for that value depended on the number (*N*) of balls intersecting at that point. For *N* = 1, the functional value of the only lesion ball that contained this point was selected as the point's value. For *N* = 2, the mean of the two values was taken. For 2 < *N* < 7, the values corresponding to all balls that contained this point were sorted in ascending order and the median value was considered to be this point's value. For *N* > 7, three median values were determined and their mean was considered to be the point's value. This algorithm (median filtering) allowed us to decrease the noise in the resulting maps. For each function, the mapped space contained 719 2D maps. Of these, 26 maps that represent the planes shown in the Paxinos and Watson (2007) atlas (12 coronal, four sagittal, and 10 transverse, or horizontal) were examined closely. The software for map generation was developed in C++. A similar technique was used earlier for producing 2D cerebellar maps for motor control impairments (Shimansky et al., 1999).

##### Statistical analyses.

Data on deep *T*_b_ (whether abdominal or colonic) and measures of thermoeffector activities (viz., HLI, VO_2_, and preferred *T*_a_) were compared between groups and across time points using two-way ANOVA with *post hoc* comparisons, as appropriate. We also identified the peak (or nadir) value of a *T*_b_ response or a thermoeffector response for each rat and compared it with the baseline value or with the corresponding value in another group using a paired or unpaired *t* test, respectively. When more than two experimental groups were compared (chemical lesions), a one-way ANOVA was used. All parametric analyses were performed only when a normal distribution of data was confirmed with a Shapiro–Wilk test. For non-normally distributed data, Wilcoxon, Mann–Whitney, rank ANOVA, and Spearman rank order correlation tests were used. All analyses were performed using SigmaPlot 11 (Systat Software). Unless stated otherwise, the data are reported as means ± SE.

#### Anatomical nomenclature

In the text and figures, all brain structures are named and abbreviated according to Paxinos and Watson (2007). In addition, we use the following three abbreviations: BNST to refer to the entire bed nucleus of the stria terminalis and PVH and DMH to refer to the relatively large areas located medially within the middle and posterior hypothalamus, respectively. Defined this way, the DMH consists of the DMC and dorsal and ventral portions (DMD and DMV, respectively) of the DM and some adjoining structures, including the DA.

## Results

### Electrolytic lesions of the DMH: gross anatomy

**Figure 1.**
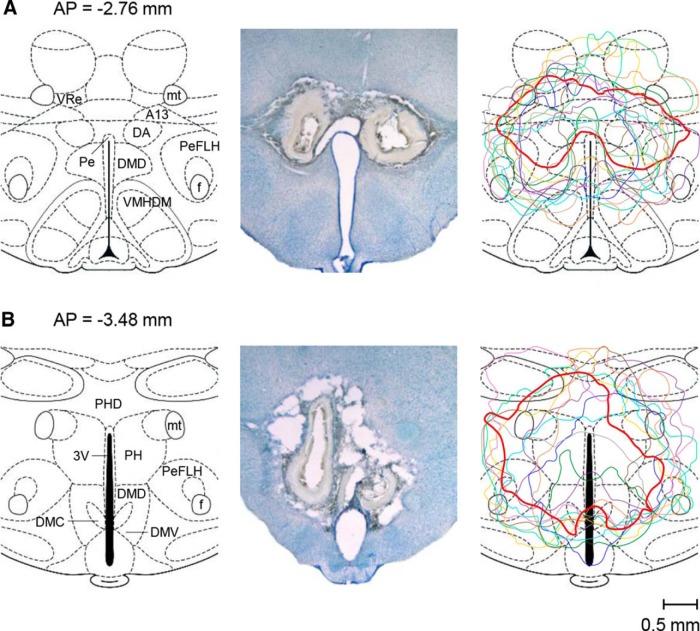
Large bilateral electrolytic lesions of the DMH in rats. The constant anodal current (1 mA, 30 s) produced lesions spreading over both the rostral (***A***) and caudal (***B***) aspects of the DMH. The two left panels show coronal hypothalamic sections (modified from Paxinos and Watson, 2007) at the specified distances from bregma (AP coordinates). The two middle panels show bright-field photomicrographs of two representative lesions in the same coronal sections, as shown on the left (cresyl violet; the scale is indicated). The two right panels contain reconstruction images (contour drawings) of nine different bilateral lesions. The two lesions shown in the photomicrographs (middle panels) are drawn with thicker red lines. 3V, Third ventricle; A13, A13 dopamine cells; PH, posterior hypothalamic nucleus; PHD, dorsal part of the posterior hypothalamic area; VRe, reuniens thalamic nucleus.

To reexamine our earlier findings (Almeida et al., 2006b) that a large bilateral electrolytic lesion of the DMH both promoted hypothermia (in response to cold exposure) and attenuated hypothermia (caused by a high dose of LPS), we produced similar lesions in 53 rats (and sham lesions in 59 rats) and then studied the effects of these lesions in greater detail. The lesions were large, typically involving both the rostral (Fig. 1*A*) and caudal (Fig. 1*B*) aspects of the DMH and spreading from the DA and dopamine cell group A13 dorsally to the dorsal aspect of the ventromedial hypothalamic nucleus (VMH) ventrally while involving the perifornical part of the lateral hypothalamus (PeFLH) laterally, as well as several other adjacent structures (Fig. 1*A*,*B*, left). As typical for this lesioning technique, the shape of an electrolytic lesion was irregular, asymmetric, and varied greatly among rats (Fig. 1*A*,*B*, middle and right) even though the same stereotaxic coordinates and lesioning parameters (current and duration) were used for all lesions. The lesioned tissue had areas of extensive gliosis and seemed to be separated from normal tissue by a wide zone of secondary damage.

### Electrolytic lesions of the DMH attenuate the autonomic defense against cold, but not against heat

**Figure 2.**
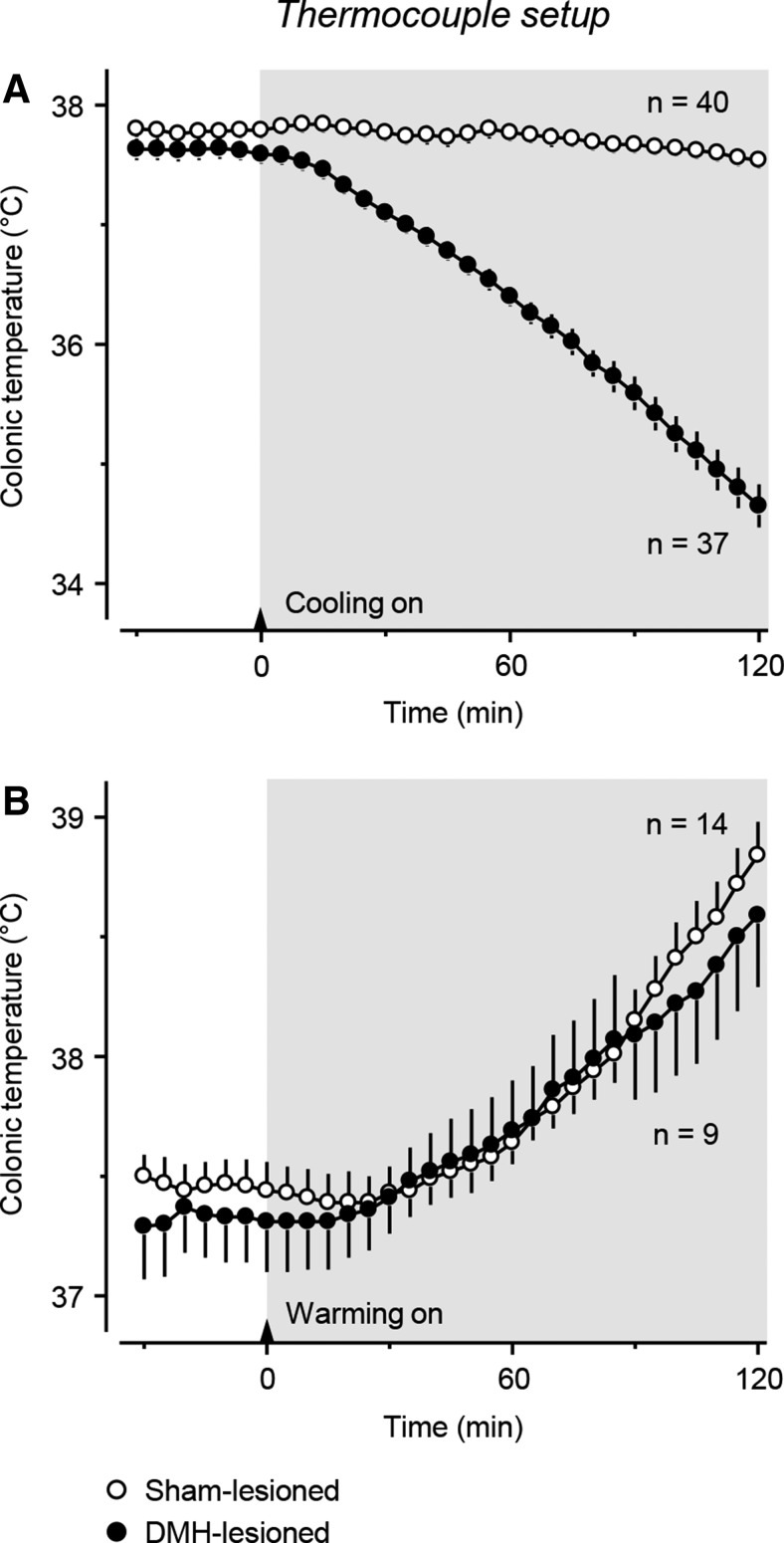
Rats with large electrolytic DMH lesions show gross insufficiency of the autonomic defense against cold (***A***), but not heat (***B***). The experiments were conducted in the thermocouple setup. Shaded areas indicate the periods of cooling (*T*_a_ decreasing from ∼28 to 10°C; ***A***) and heating (*T*_a_ increasing from ∼26 to 32°C; ***B***), which started at 0 (arrows) and lasted for 120 min. The number of rats in each experimental group (*n*) is indicated.

In response to cold exposure in the thermocouple setup (*T*_a_ decreasing from 28.3 ± 0.1 to 10.0 ± 0.2°C over 120 min), sham-lesioned rats successfully defended their *T*_b_, whereas DMH-lesioned rats failed to do so (lesion type × time interaction: *F*_(1,2250)_ = 145.12; *p* < 0.001; Fig. 2*A*). At the end of the 2 h exposure, the difference in *T*_b_ between sham- and DMH-lesioned rats was ∼2.9°C (*p* < 0.001). We then tested how rats were able to defend their *T*_b_ against heat (*T*_a_ increasing from 25.8 ± 0.1 to 32.0 ± 0.1°C over 120 min). Large electrolytic DMH lesions did not weaken the autonomic defense of *T*_b_ against heat (lesion type × time interaction: *F*_(1,630)_ = 0.59; *p* = 0.962; Fig. 2*B*). At the end of the 2 h heat exposure, the increase in *T*_b_ in the DMH-lesioned rats (1.3 ± 0.2°C) was nearly identical to that in the sham-lesioned rats (1.4 ± 0.2°C; *p* = 0.701). A selective effect of large DMH lesions on the autonomic defense against cold, but not against heat, was observed by Almeida et al. (2006b). Importantly, in the heating experiment (Fig. 2*B*), the initial *T*_a_ was relatively low (∼26°C) and the baseline *T*_b_ in this cool environment was ∼0.2°C lower in DMH-lesioned rats than in sham-operated controls (*p* = 0.073). Low values of *T*_b_ in DMH-lesioned rats exposed to room temperature were also reported by others (Chou et al., 2003; Gooley et al., 2006).

### Electrolytic lesions of the DMH attenuate LPS-induced cold-seeking behavior and hypothermia, but not LPS-induced warmth-seeking behavior or fever

**Figure 3.**
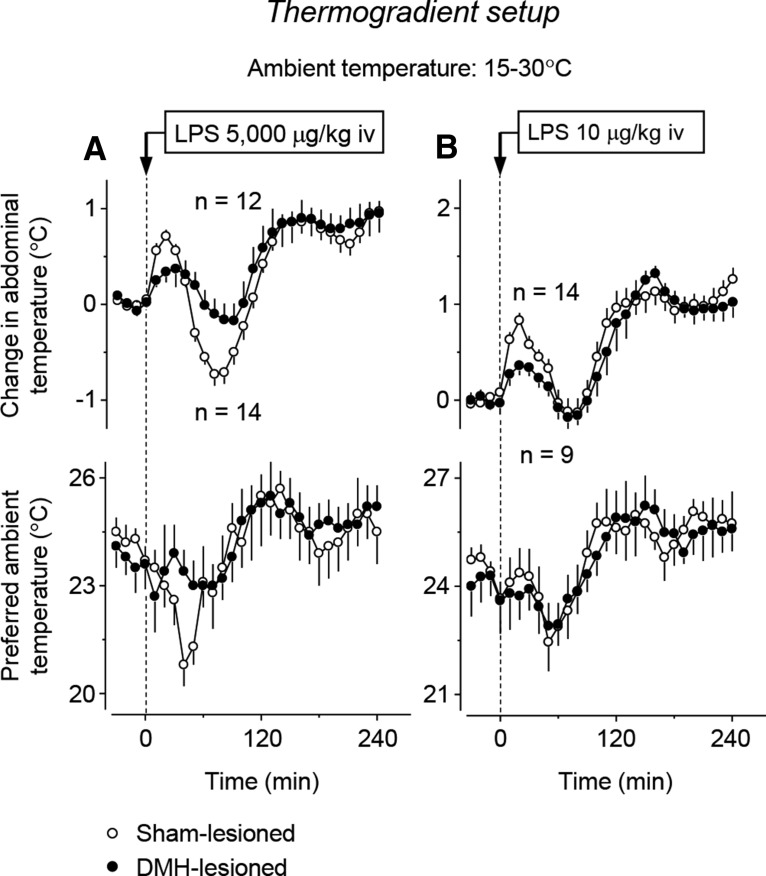
Electrolytic DMH lesions block hypothermia and cold-seeking behavior induced by the high dose of LPS (5000 μg/kg, i.v.; ***A***), but not fever or warmth-seeking behavior induced by the low dose (10 μg/kg, i.v.; ***B***), in rats freely moving in a thermogradient apparatus. Baseline values for abdominal *T*_b_ of DMH-lesioned rats were as follows: 36.2 ± 0.2°C (high dose of LPS) and 36.4 ± 0.2°C (low dose). Baseline values for *T*_b_ of sham-lesioned rats were as follows: 36.5 ± 0.1°C (high dose) and 36.6 ± 0.1°C (low dose). Please note that here and in Figure 4, the nadirs and peaks of the curves are not the same as the mean maximum and minimum values of the corresponding variables listed in the text because the maxima and minima were reached by different rats at different times.

We then tested the ability of DMH-lesioned rats to develop hypothermia and cold-seeking behavior in the thermogradient setup in response to a high, shock-inducing dose of LPS (5000 μg/kg, i.v.) (Fig. 3*A*). Whereas sham-lesioned rats displayed strong cold-seeking behavior (a 4.8 ± 0.7°C maximum decrease in preferred *T*_a_ at 47 ± 3 min post-LPS; *p* < 0.001), which was coupled with a 0.9 ± 0.1°C decrease in abdominal *T*_b_ (nadir at 69 ± 3 min; *p* < 0.001), both responses were strongly attenuated in DMH-lesioned rats. Lesioned rats exhibited a 1.8 ± 1.2°C maximum decrease in preferred *T*_a_ (at 44 ± 5 min; *p* = 0.033 for the lesion–sham difference) and a 0.4 ± 0.2°C decrease in abdominal *T*_b_ (at 69 ± 3 min; *p* = 0.011 for the lesion–sham difference). In response to a high dose of LPS, the hypothermic response is often followed by fever (Almeida et al., 2006a; Steiner and Romanovsky, 2015); the same pattern was observed in the present study (Fig. 3*A*). The fever portion of the response was associated with warmth-seeking behavior (a 4.8 ± 0.6°C maximum increase in preferred *T*_a_ at 126 ± 11 min post-LPS; *p* < 0.001). DMH lesioning did not affect this part of the response (3.7 ± 1.3°C maximum increase in preferred *T*_a_ at 132 ± 17 min post-LPS; *p* = 0.391).

In a separate experiment, rats were injected with a low, pyrogenic dose of LPS (10 μg/kg i.v.), which causes fever and warmth-seeking behavior (Almeida et al., 2006a). In the present study, this dose also triggered warmth-seeking behavior (preferred *T*_a_ increased by 4.3 ± 0.7°C at 143 ± 20 min post-LPS; *p* < 0.001) and fever (*T*_b_ increased by 1.5 ± 0.1°C at 190 ± 12 min; *p* < 0.001) without causing hypothermia (Fig. 3*B*). Neither the warmth-seeking behavior (preferred *T*_a_ increased by 5.5 ± 1.1°C at 149 ± 29 min post-LPS) nor fever (*T*_b_ increased by 1.6 ± 0.1°C at 162 ± 12 min) was affected by DMH lesioning (*p* = 0.383 and 0.257, respectively; lesion–sham differences). Saline-treated controls, whether sham-lesioned or DMH-lesioned, exhibited no marked changes in either their preferred *T*_a_ or deep *T*_b_ (data not shown).

In this experiment, LPS (Fig. 3*A*,*B*) or saline (data not shown) was administered as a bolus infusion through the preimplanted jugular catheter. Each rat was briefly restrained by hand in its channel of the thermogradient apparatus. This procedure caused a small and short-lasting (but reproducible) hyperthermic response. In saline-treated sham-lesioned rats, this stress hyperthermia reached a peak of 1.1 ± 0.1°C at 14 ± 2 min (*p* < 0.001). Under the same conditions, DMH-lesioned rats exhibited blunted stress hyperthermia with a maximum *T*_b_ increase of only 0.6 ± 0.1°C (*p* < 0.003).

Therefore, by conducting a large number of experiments in a slightly different paradigm and with longer recording times, we were able to reproduce the two phenomena discovered by Almeida et al. (2006b): (1) the suppression of LPS-induced cold-seeking behavior and of hypothermia (but not of LPS-induced warmth-seeking behavior or fever) and (2) the suppression of autonomic cold defense by DMH ablation. It was imperative to do so because both phenomena have only been demonstrated in that single study. As a new observation, we now show a large DMH lesion attenuates stress hyperthermia (the hyperthermic response to handling). After confirming that a large bilateral electrolytic lesion of the DMH both promotes hypothermia (in response to cold exposure) and attenuates hypothermia (caused by a high dose of LPS), we attempted to reconcile these seemingly contradicting findings by examining their thermoeffector patterns and neuroanatomic substrates.

### Autonomic effectors are not involved in the attenuation of LPS hypothermia by DMH lesions

In our experiments, the response to cold exposure was studied in rats that were confined to their restrainers and could not use behavioral thermoregulation. Therefore, the hypothermia-promoting effect of DMH lesions in this setup was due to an effect on the autonomic cold defense. Conversely, the response to LPS was studied in the thermogradient setup, where rats could use both behavioral and autonomic effectors to regulate their *T*_b_. In this setup, DMH lesions blocked cold-seeking behavior, but whether they also affected autonomic effectors was not tested.

**Figure 4.**
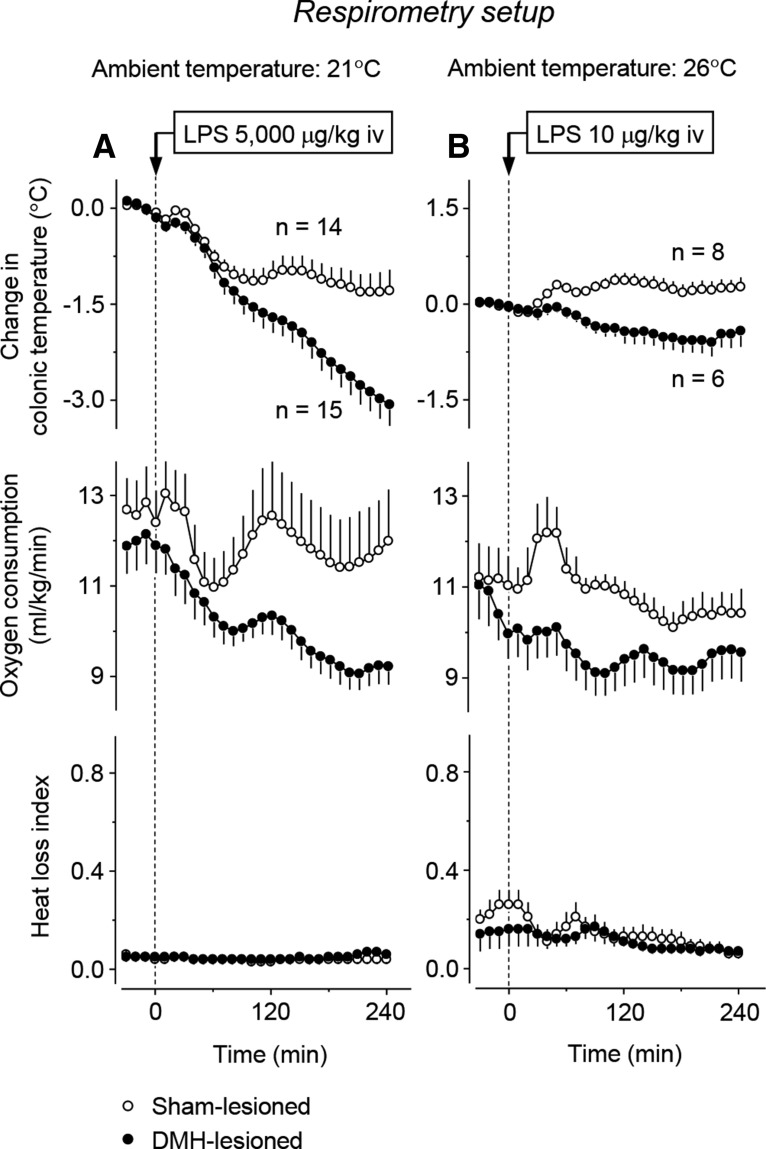
Thermoeffector pattern of the responses of DMH-lesioned and sham-lesioned restrained rats to LPS in the respirometry setup. The level of thermogenesis in DMH-lesioned rats is always lower than in sham-lesioned rats. The high dose of LPS (5000 μg/kg, i.v.) was administered at a subneutral *T*_a_ (21°C; ***A***); the low dose (10 μg/kg, i.v.) was administered at a neutral *T*_a_ (26°C; ***B***). Baseline values for colonic *T*_b_ of DMH-lesioned rats were as follows: 37.1 ± 0.2°C (high dose of LPS) and 37.4 ± 0.1°C (low dose). Baseline values for *T*_b_ of sham-lesioned rats were as follows: 37.3 ± 0.1°C (high dose) and 37.5 ± 0.2°C (low dose).

To test whether an impairment of autonomic thermoeffectors also contributes to the inability of DMH-lesioned rats to develop hypothermia in response to the high dose of LPS, we studied the *T*_b_, VO_2_, and HLI of restrained rats in the respiratory setup. Two experiments were performed. The first experiment was conducted at a subneutral *T*_a_ of 21°C (Table 1). At this *T*_a_, sham-lesioned rats responded to the high dose of LPS with hypothermia (a maximum *T*_b_ decrease of 1.7 ± 0.3°C at 158 ± 18 min; *p* < 0.001) and a 21% drop in metabolic rate (a maximum VO_2_ decrease of 3.1 ± 0.6 ml/kg/min at 111 ± 21 min; *p* < 0.001; Fig. 4*A*). In DMH-lesioned rats, both the hypothermic response and the decrease in metabolism were not attenuated, but markedly exaggerated (lesion type × time interaction: *F*_(1,729)_ = 10.11 and 3.70, respectively; *p* < 0.001 for both). At 4 h after LPS administration, the *T*_b_ of DMH-lesioned rats was 2°C lower than in controls (34.0 ± 0.4°C vs 36.0 ± 0.3°C; *p* < 0.001). At the same time, VO_2_ values in DMH-lesioned rats were suppressed by 24% compared with sham-lesioned controls (12.5 ± 1.1 vs 9.5 ± 0.4 ml/kg/min; *p* = 0.015). Because the experiments were performed at a subneutral *T*_a_, all rats (whether DMH lesioned or sham lesioned) exhibited continuous tail-skin vasoconstriction throughout the experiment (near zero HLI values). These results show that, when rats cannot regulate their *T*_b_ behaviorally, DMH lesions do not block LPS-induced hypothermia. These results further suggest that the attenuation of LPS hypothermia observed in the thermogradient setup was not due to an effect on thermogenesis because the LPS-induced decrease in thermogenesis is not attenuated, but rather exaggerated, by DMH lesions.

Because the thermoregulatory response to LPS strongly depends on both LPS dose and *T*_a_, we performed a second experiment in the respirometry setup. This was conducted at a neutral *T*_a_ of 26°C (Table 1) and involved the low dose of LPS (10 μg/kg, i.v.). In response to this dose, sham-lesioned rats developed a low-grade fever: at 120 min, their *T*_b_ increased by 0.4 ± 0.1°C relative to baseline values (*p* = 0.050; Fig. 4*B*). The effector pattern of this response included a short-lived burst in the metabolic rate (an ∼15% increase in the peak VO_2_ at 40 ± 3 min; *p* < 0.001) and two phases of cutaneous vasoconstriction. The vasoconstriction response was masked by the fact that the sham-lesioned rats were already vasoconstricted (i.e., had a low HLI) at the time of LPS administration; nevertheless, the observed additional decrease in the HLI was statistically significant (*p* = 0.028). These febrile (lesion type × time interaction: *F*_(1,324)_ = 7.19; *p* < 0.001) and thermogenic (lesion type × time interaction: *F*_(1,324)_ = 2.01; *p* = 0.002) responses to LPS were completely blocked in DMH-lesioned rats. The tail-skin vasoconstriction response did not occur in DMH-lesioned rats either, but the tails of DMH-lesioned rats already showed strong vasoconstriction at the time of LPS administration. Similar to the experiment with the high dose of LPS conducted at a subneutral *T*_a_, the experiment with the low dose of LPS administered at a neutral *T*_a_ did not reveal any autonomic thermoeffector response that could have contributed to the attenuation of LPS-induced hypothermia by DMH lesions observed in experiments in the thermogradient setup. Therefore, our present and previous (Almeida et al., 2006b) studies dealt with two distinct, unrelated effects of large electrolytic DMH lesions: on thermogenesis (attenuation of the thermogenic response to cold, LPS, and perhaps stress) and on behavior (attenuation of the cold-seeking response to high doses of LPS). We then proposed that the two effects were due to lesioning of different sites within the DMH and attempted to identify the responsible sites.

### Thermal lesions within the DMH: gross anatomy

**Figure 5.**
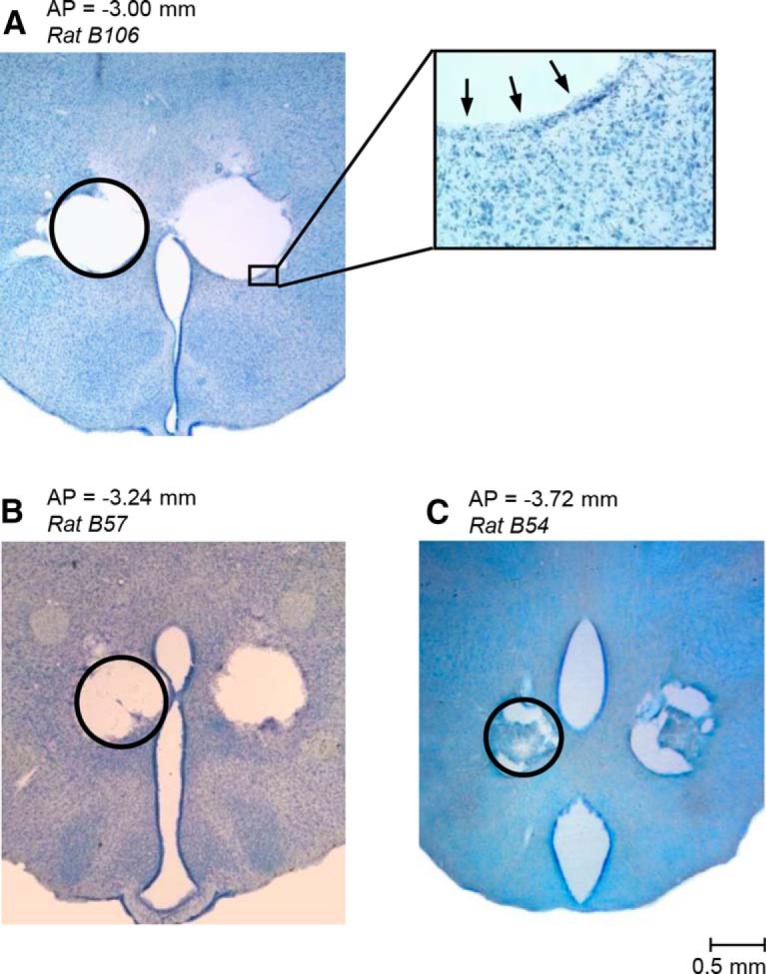
Representative bilateral thermal lesions in the rostral (***A***), central (***B***), and caudal (***C***) aspects of the DMH. To produce a lesion, the brain tissue was heated locally to 70–75°C with alternating (radiofrequency) current. Most lesions have a near-round shape (circles are drawn for comparison) and a well defined, narrow demarcation zone (arrows) without marked secondary tissue damage beyond this zone. Cresyl violet staining, the AP coordinates (Paxinos and Watson, 2007), and the scale are indicated.

To produce smaller and better-delineated DMH lesions, we used the technique of thermal coagulation by alternating (radiofrequency) current. We wanted to place small symmetric bilateral lesions that cumulatively would cover the entire area of the large electrolytic lesions described above (Fig. 1). Therefore, for thermal lesions, we always used the same stereotaxic coordinates on the left and right sides, but varied the coordinates between rats (see “Thermal brain tissue lesioning” section); 83 DMH-lesioned rats (and 10 sham-lesioned rats) were used. Examples of three pairs of lesions (in three rats) are shown in Figure 5. In drastic contrast to electrolytic lesions, thermal lesions had a clearly defined, regular, near-round shape with a diameter ranging from 300 to 1000 μm. All lesions passed a test for symmetry: a pair of lesions was considered acceptable when each lesion, in the section that included its center, overlapped by >50% (area) with the mirror image of the lesion from the opposite side. The demarcation zone between the missing tissue and normal tissue for thermal lesions was narrow and the basic histological examination of cresyl violet-stained sections revealed no obvious changes in brain tissue characteristics beyond this narrow zone.

### Effects of thermal lesions within the DMH on the autonomic cold defense and LPS-induced cold-seeking behavior

**Figure 6.**
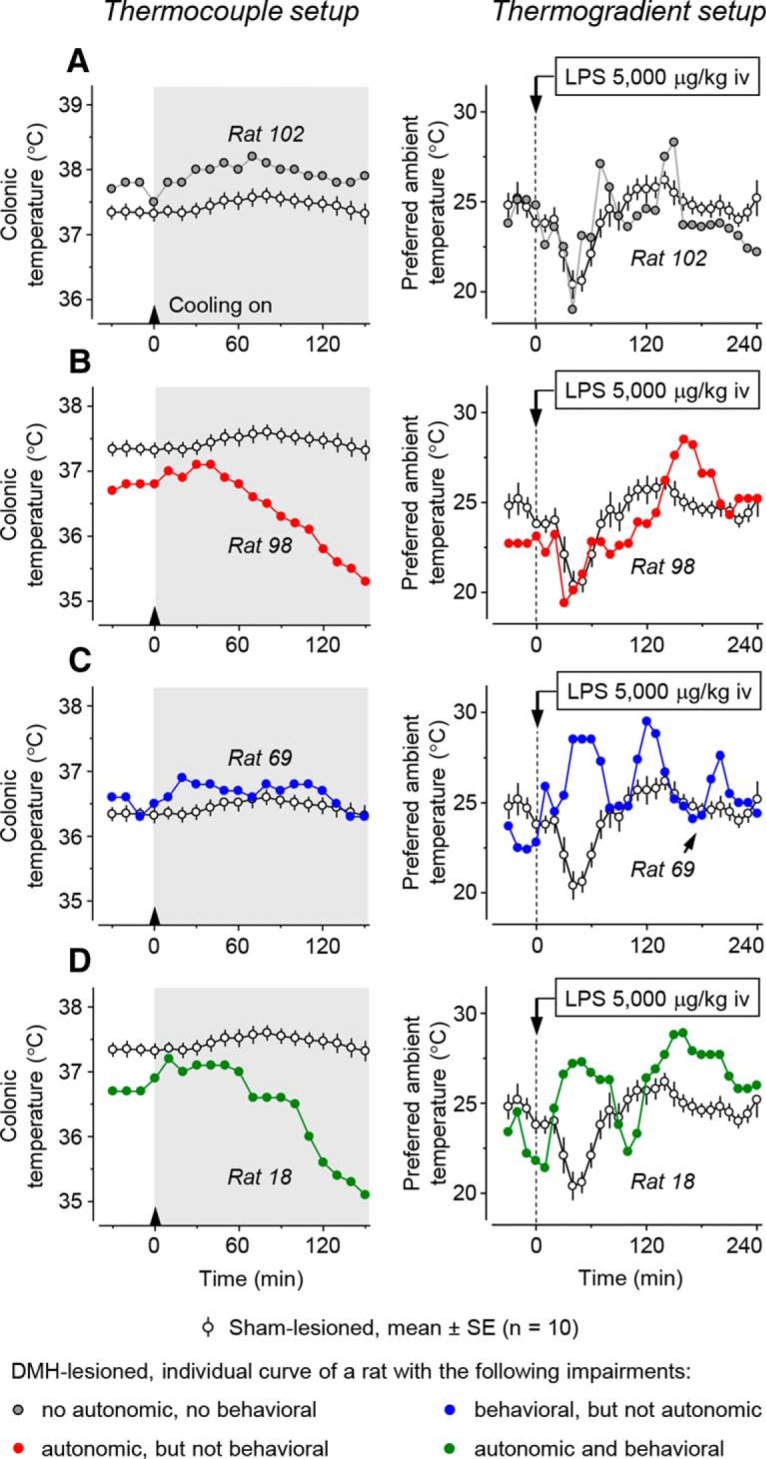
The effects of a bilateral thermal lesion within the DMH on the autonomic cold-defense response and on the cold-seeking behavior associated with systemic inflammation. Left, Autonomic effect. In each panel, the colonic *T*_b_ response of a DMH-lesioned rat to external cooling (a *T*_a_ decrease from ∼28 to 9°C over 150 min) is shown and compared with the mean response of 10 sham-lesioned rats. Right, Behavioral effect. In each panel, the preferred *T*_a_ response of a DMH-lesioned rat to a shock-inducing dose of LPS (5000 μg/kg, i.v.) is shown and compared with the mean response of 10 sham-lesioned rats. The four outcome combinations were observed: no impairment found by either test (***A***); an impaired autonomic defense of *T*_b_ against cold without impairment of the cold-seeking response to the high dose of LPS (***B***); an impaired LPS-induced cold-seeking response but no impairment of the autonomic cold defense (***C***); and impairments of both the autonomic defense against cold and the cold-seeking response to LPS (***D***).

Each rat with a bilateral thermal lesion was subjected to two tests. In the first test, we exposed it to cold (*T*_a_ decreasing from 28.0 ± 0.0 to 8.8 ± 0.2°C over 150 min) in the thermocouple setup, where it could not use behavioral thermoregulation. We then compared the *T*_b_ response of each DMH-lesioned rat (individual response) with the mean *T*_b_ response of 10 sham-lesioned rats (Fig. 6). In the second test, we studied the cold-seeking behavior in response to LPS (5000 μg/kg, i.v.) in the thermogradient apparatus, where a rat could select its preferred thermal environment. The preferred *T*_a_ response of each DMH-lesioned rat (individual response) was then compared with the mean preferred *T*_a_ response of 10 sham-lesioned rats. Figure 6 shows the individual responses of four DMH-lesioned rats, compares them with the mean responses of 10 sham-lesioned rats, and illustrates four different outcomes. These outcomes are as follows: (1) the lesion caused no autonomic impairment (did not block or attenuate the autonomic cold defense) and no behavioral impairment (did not block or attenuate LPS-induced cold-seeking behavior) (Fig. 6*A*); (2) the lesion caused an autonomic impairment (blocked or attenuated the autonomic cold defense), but no behavioral impairment (Fig. 6*B*); (3) the lesion caused a behavioral impairment (blocked or attenuated LPS-induced cold-seeking behavior), but no autonomic impairment (Fig. 6*C*); and (4) the lesion caused both impairments (Fig. 6*D*). These experiments clearly showed that the two impairments observed (autonomic and behavioral) were independent of each other.

### DMH representation of the autonomic and behavioral impairments: preliminary analysis

We then investigated whether the two impairments were caused by lesioning different sites within the DMH. For each lesioned rat, we calculated two indices: the index of autonomic cold defense impairment and the index of impairment of LPS-induced cold-seeking behavior (see “Data processing and analysis” section). In brief, the index of autonomic impairment was calculated as the relative decrease in colonic *T*_b_ of the rat of interest compared with the mean decrease of 10 sham-lesioned rats. For a rat with a strong autonomic impairment (compromised ability to defend *T*_b_ against cold; see Fig. 6*B*,*D*), the impairment index is a high (up to 5°C) positive number. If there is no impairment, then the index has a near zero value, either positive or negative (Fig. 6*A,C*). The behavioral impairment index was calculated as the relative increase in the preferred ambient temperature of a lesioned rat after LPS administration (compared with the mean of 10 sham-lesioned rats). For a rat with a strong behavioral impairment (compromised ability to decrease the preferred *T*_a_ in response to the high dose of LPS; Fig. 6*C,D*), the impairment index is a high (up to 8°C) positive number. If there is no impairment, the index has a near zero value (Fig. 6*A,B*).

**Figure 7.**
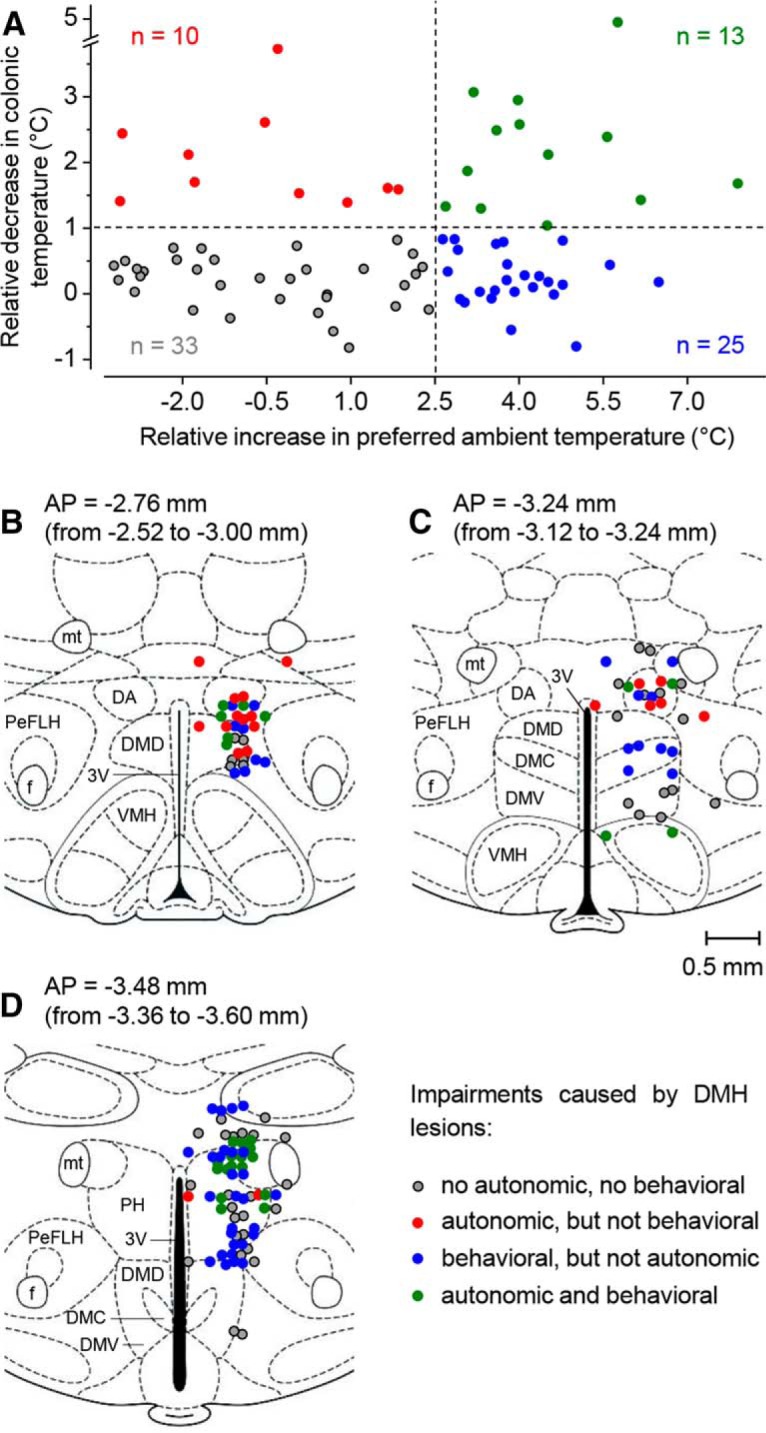
Preliminary analysis of the DMH representation of autonomic and behavioral impairments. The four possible combinations of the two impairments (Fig. 6) are shown by plotting the autonomic impairment index (the relative decrease in colonic *T*_b_) against the behavioral impairment index (the relative increase in preferred *T*_a_) (***A***). The location of lesion centers corresponding to the four outcome combinations is shown for the three thick coronal sections of the DMH: from 2.52 to 3.00 mm caudal to bregma (***B***); from 3.12 to 3.24 mm caudal to bregma (***C***); and from 3.36 to 3.60 mm caudal to bregma (***D***). The number of rats (each with a bilateral DMH lesion) is shown. All abbreviations of the names of brain structures are according to Paxinos and Watson (2007) and are the same as in Figure 1.

We then plotted the two indices for each rat against each other (Fig. 7). To be on the conservative side, we selected relatively high thresholds for the autonomic and behavioral impairments: 1.0 and 2.5°C, respectively. The higher threshold for behavioral impairment was chosen because the preferred *T*_a_ varies in a much wider range than deep *T*_b_. This plot allowed us to reveal the same four experimental outcomes as shown in Figure 6.

We then mapped lesions that caused different effects (Fig. 7*B–D*). In this preliminary analysis, the coordinates of the lesion center were determined by averaging the coordinates for the right and left lesions and each rat was represented by a single set of coordinates. This analysis revealed that the autonomic impairment was caused by lesions in and around the DA (red and green symbols in Fig. 7*B*,*C*). Five rats with the most compromised ability to defend *T*_b_ autonomically against cold had the mean autonomic impairment index of 3.6 ± 0.3°C and the following mean coordinates of the lesion center: AP = −3.03 ± 0.11 mm; ML = 0.58 ± 0.05 mm; and DV = −8.26 ± 0.04 mm. The point with these coordinates belongs to the DA. Compared with the autonomic impairment, the behavioral impairment was caused by lesions located more caudally (compare the ratio between the number of blue to red symbols in Fig. 7*B–D*) and more ventrally (compare the distribution of blue and red symbols in Fig. 7*C*). This impression, however, was not confirmed definitively when we looked at the five rats with the most compromised ability to respond to the high dose of LPS with cold-seeking behavior (behavioral impairment index of 6.4 ± 0.4°C). The mean coordinates of lesion center for these rats were as follows: AP = −3.37 ± 0.16 mm; ML = 0.72 ± 0.06 mm; and DV = −8.34 ± 0.21 mm.

We have also established that the impairments caused by the lesions did not depend on the extent of brain tissue damage. Neither the autonomic impairment (*r* = 0.051; *p* = 0.650; Spearman rank test) nor the behavioral one (*r* = −0.129; *p* = 0.250) correlated significantly with the lesion diameter. Therefore, the differences in effects caused by various lesions were not due to lesion sizes, but rather lesion locations.

### DMH topography of the autonomic defense against cold and of LPS-induced cold-seeking behavior

**Figure 8.**
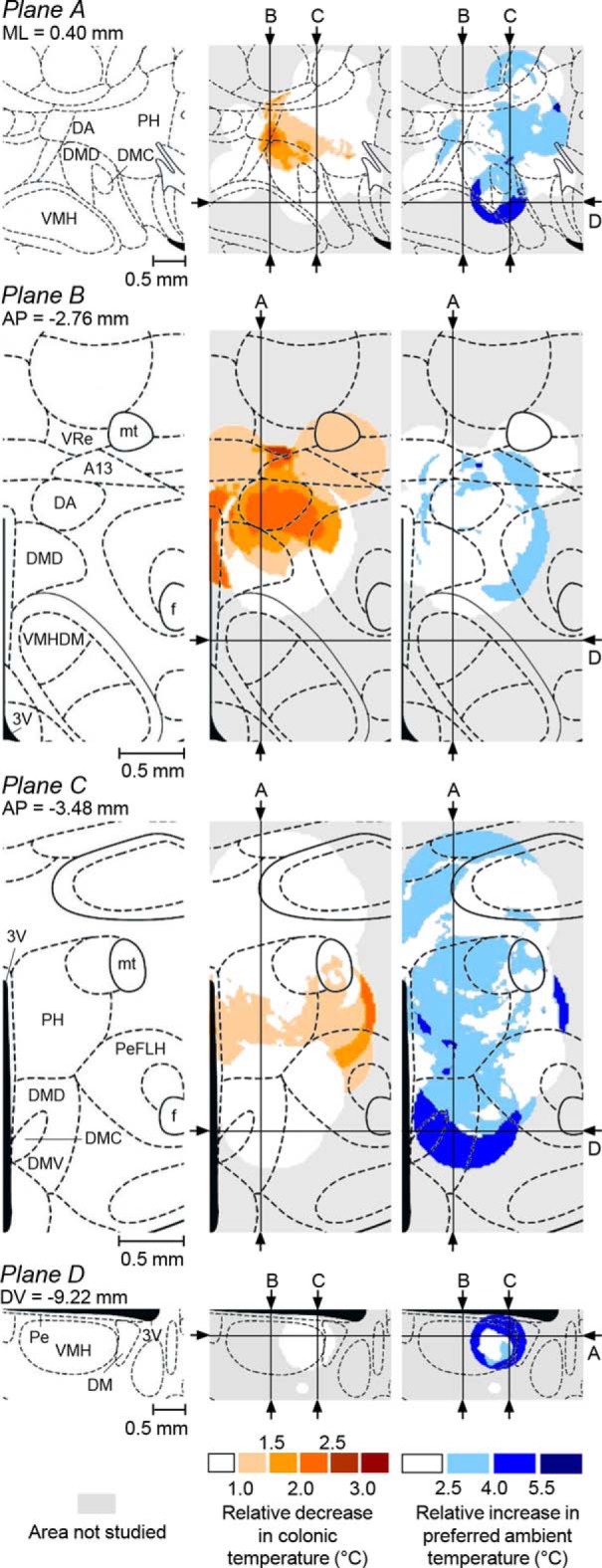
Autonomic cold defense and LPS-induced cold-seeking behavior: representation maps. Left, Sagittal section (top row; Plane A), two coronal sections (second and third rows; Planes B and C), and a transversal DMH section (bottom row; Plane D) at the specified coordinates (modified from Paxinos and Watson, 2007). The middle and right columns show the corresponding representation maps for the autonomic cold defense and for LPS-induced cold-seeking behavior, respectively. In each map, arrows and letters indicate the lines at which the plane of this map is crossed by all other planes shown. The magnitude of the autonomic and behavioral impairments used for constructing these maps is coded by color. All abbreviations of the names of brain structures are according to Paxinos and Watson (2007) and are the same as in Figures 1 and 7.

We built detailed representation maps to localize more precisely the unknown site that drives cold-seeking behavior in severe systemic inflammation (and, as a control, the known site that drives cold-induced thermogenesis) in 3D space. Our procedure (see “Construction of functional representation maps” section) accounted, not only for the lesion location, but also for the lesion size, as well as for the magnitude of impairment at each location. For each impairment studied, 26 maps were examined closely and four maps are shown in Figure 8. The top row of images includes a schematic of the sagittal brain section (0.40 mm from the midline; Plane A) and the corresponding representation maps for the two functions of interest. For this plane, the first map shows that the autonomic cold defense (thermogenesis) critically depends on a relatively small (∼0.4 × 0.5 mm) area of darker colors in the ventrocaudal aspect of the DA and the most dorsal aspect of the DMD. The second map shows that the cold-seeking behavior critically depends on the site located ∼0.5 mm caudally and ∼0.5 ventrally from the site that controls thermogenesis. In Plane A, the projection of this site has the shape of a crescent but, cumulatively, all maps presented in Figure 8 indicate that the 3D shape of this site is a hemisphere of ∼1 mm in diameter, which sits on top of the caudal aspect of the VMH like a bowl and supports/contains the central aspect of the DM (the DMD–DMC–DMV complex). We then made two coronal sections through the centers of the two sites, Planes B and C. The first section (Plane B, 2.76 mm caudal to bregma) contains the thermogenesis-driving site, whereas the second section (Plane C, third row, 3.48 mm caudal to bregma) contains the site that drives cold-seeking behavior. In Plane B, the thermogenesis-driving site closely corresponds to the DA, with limited spreading into the surrounding structures: the medial aspect of A13 and the most ventral aspect of the reuniens thalamic nucleus, dorsally; the dorsal aspect of the periventricular nucleus (Pe), medially; the most dorsal aspect of the DMD, ventrally; and the dorsomedial aspect of the PeFLH, laterally. In Plane C, cold-seeking behavior has the highest representation (dark blue) in the ventral aspect of the DM (involving all three subdivisions: the DMD, DMC, and DMV) and extends laterally toward the PeFLH. The bottom row of images (Plane D) shows the cold-seeking behavior-driving site as a ring, which represents the “wall or the bowl” sitting on top of the VMH. In this plane, the site extends into the DMD caudally and into the Pe medially.

**Figure 9.**
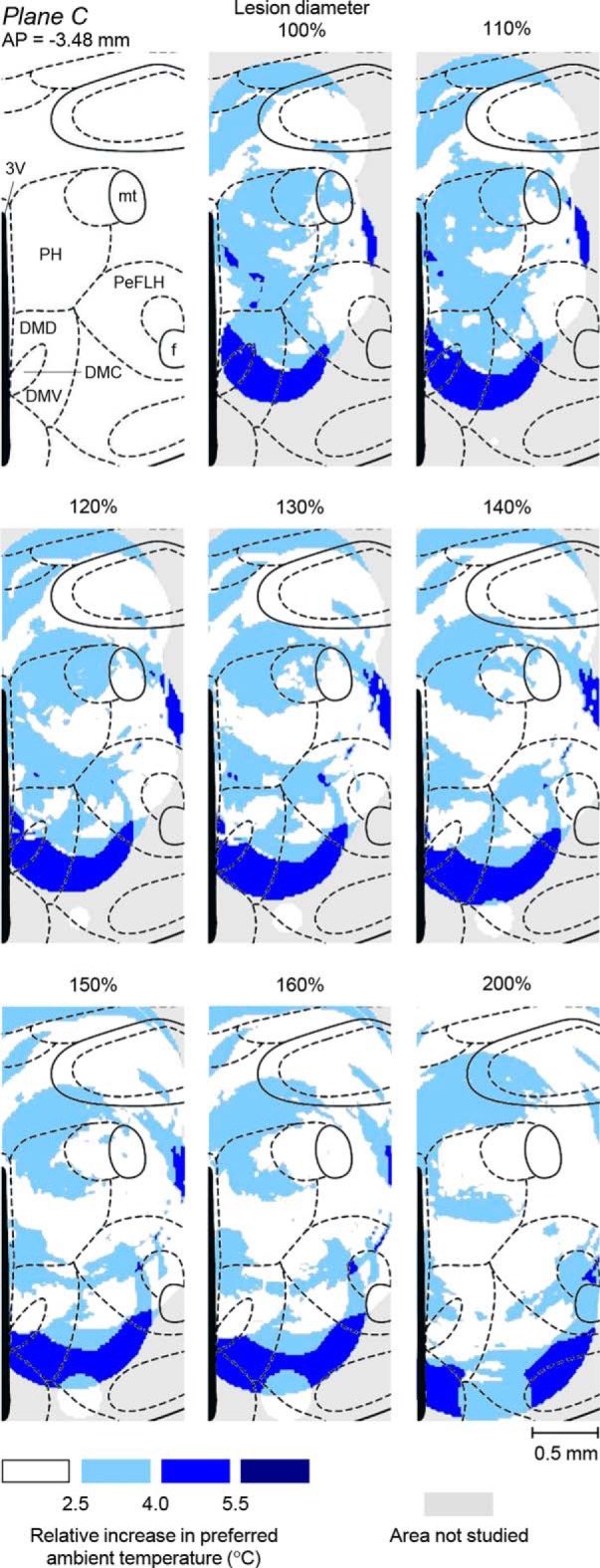
The effects of increasing the diameter of thermal lesions on the size, shape, and location of the DMH representation site for LPS-induced cold-seeking behavior. The left panel in the top row shows a coronal DMH section at 3.48 mm caudal to bregma (modified from Paxinos and Watson, 2007). The middle panel in the top row shows a representation map for LPS-induced cold-seeking behavior, which was constructed assuming that the area of lost function for each thermal lesion equaled the area of lost tissue (lesion diameter, 100%); the same map is shown in Figure 8. Other panels show how this functional topography would change if we were to use larger diameters (110–200%) for all thermal lesions. All abbreviations of the names of brain structures are the same as in Figures 1, 7, and 8.

The diameters of the thermal lesions were determined based on the area lacking neuronal tissue (Fig. 5*A*). However, the area of lost tissue might have been separated from normal, fully functional tissue with a zone of functional deficiency (e.g., due to inflammation around the lesion), thus making the “functional lesion” area greater than the area of missing tissue. Therefore, we might have underestimated the size of thermal lesions and this might have distorted the representation maps. To test how sensitive our maps were to the lesion size, we computed an additional series of maps in which the diameter of each lesion was increased by 10–100% over the measured diameter (Fig. 9). This series demonstrates that our maps are reasonably robust because the location, shape, and size of the site that drives cold-seeking behavior do not change much in the range 100–140% of the measured lesion diameter. With a further increase in the lesion diameter, the site slowly shifts in the ventral direction and extends laterally until it starts disintegrating at a diameter of 200% (which corresponds to a lesion volume of 800% of the measured volume of lost tissue). The demonstrated robustness of the map warrants that the determined location and size of the site that drives cold-seeking behavior in severe systemic inflammation are reasonably accurate even if we somewhat underestimated the size of “functional lesions.”

### LPS-induced cold-seeking behavior, but not the autonomic defense of *T*_b_ against cold, critically depends on neuronal bodies at the identified site

**Figure 10.**
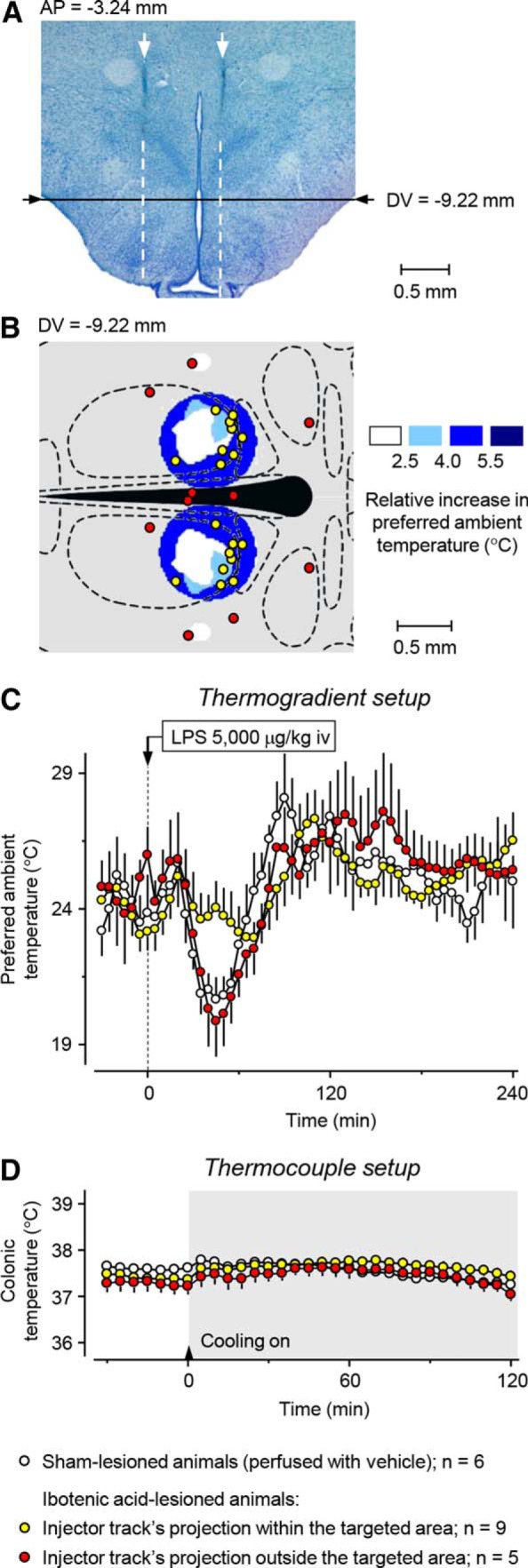
Excitotoxic (ibotenic acid) lesions of the newly defined site at the DM–VMH border block LPS-induced cold-seeking behavior, but do not affect the autonomic defense of *T*_b_ against cold. A representative photomicrograph of a rat brain with a bilateral lesion induced by ibotenic acid shows that the lesion center on each side was determined as the intersection point of a straight-line extension (white dashed line) of the injector cannula track (white arrow) and the −9.22 mm (DV) transverse plane (Paxinos and Watson, 2007) (***A***). Of 14 rats with excitotoxic lesions, 9 (yellow circles) had lesion centers within the targeted site (colored with dark blue) and five (red circles) had lesion centers outside this site, as shown on the schematic of the −9.22 mm (DV) transverse brain section (***B***). Sham-lesioned rats (white circles) and rats with lesion centers outside the targeted site showed typical cold-seeking behavior in response to the high dose of LPS (5000, μg/kg i.v.), whereas rats with lesion centers within the targeted site did not select a lower *T*_a_ in response to LPS (***C***). Sham-lesioned rats and rats with excitotoxic lesions, regardless of their location, were all able to defend successfully their *T*_b_ autonomically against environmental cooling (from ∼28 to 7°C) in the thermocouple setup (***D***).

We have shown previously that the ability of rats to respond to a high dose of LPS with cold seeking can be impaired not only by large electrolytic lesions, but also by large excitotoxic (ibotenic acid) lesions of the DMH (Almeida et al., 2006b). This indicates that LPS-induced cold-seeking behavior critically depends on neuronal bodies (rather than fibers of passage) somewhere within the DMH. Based on the constructed functional maps (Fig. 8), we determined the stereotaxic coordinates of this neuronal group as follows: AP, 2.90–3.80 mm caudal to bregma; ML, 0.10–0.90 mm from the midline; and DV, 8.90–9.50 mm below bregma (Fig. 8). To confirm the location and response specificity of the neuronal group that drives cold-seeking behavior in systemic inflammation (as revealed by our mapping approach), we infused ibotenic acid into the newly identified site in 14 rats; six additional rats were used as sham-lesioned controls. To produce smaller excitotoxic lesions, we used the same concentration of ibotenic acid as in the Almeida et al., 2006b study (10 ng/nl), but decreased the volume from 300 to 75 nl. At this concentration, ibotenic acid causes neuronal loss, as we confirmed histologically (Almeida et al., 2006b), but the area of loss cannot be clearly delineated at a lower magnification. At a higher magnification, at which the border between lesioned and intact tissue can be determined more reliably, the lesion spans over multiple visual fields and it becomes difficult to determine the lesion center in a reproducible way. To circumvent this problem, we identified the lesion center by extending the injector cannula track with a straight line and finding a point at which this line intersected the −9.22 mm (DV) transverse plane (Paxinos and Watson, 2007), as illustrated in Figure 10*A*. This way, the two coordinates that vary the most due to any imperfection in the stereotaxic injection procedure and individual variations between rats (i.e., the AP and ML coordinates) were determined directly based on the injector track, whereas the third, least likely to vary coordinate (DV) was determined by adding 0.22 mm to the depth to which the injector cannula tip was inserted (9.00 mm below bregma). We then plotted all lesion centers on the −9.22 mm transverse plane and separated the animals into two groups: those that had the centers of excitotoxic lesions on both sides of the brain within (yellow circles) and outside (red circles) the targeted site (Fig. 10*B*). The rats that had excitotoxic lesions outside the targeted site and also sham-lesioned rats responded to the shock-inducing dose of LPS with marked cold-seeking behavior (an ∼5°C decrease in the preferred *T*_a_ in the thermogradient apparatus; *p* = 0.007 and *p* = 0.028, respectively), whereas the rats that had excitotoxic lesions within the targeted site did not develop cold-seeking behavior (Fig. 10*C*). During the time period of 40–50 min after LPS administration, these rats' thermopreferendum was 2.4°C higher on average than that of the sham-lesioned rats (*p* < 0.05). Conversely, there was no difference in the autonomic response to environmental cooling (a decrease in the *T*_a_ from 28.2 ± 0.1 to 7.3 ± 0.2°C over 120 min) between rats with excitotoxic lesions within the targeted site versus outside the targeted site versus with sham lesions (*F*_(2,570)_ = 0.53, *p* = 0.596; Fig. 10*D*). All three groups of rats were able to defend their *T*_b_ autonomically, confirming that the DMH site essential for LPS-induced cold-seeking behavior is different from the DA site that drives thermogenesis.

## Discussion

### Thermoregulatory effects of large DMH lesions

In our earlier (Almeida et al., 2006b) and present studies, restrained rats with large electrolytic lesions of the DMH responded to cold exposure with hypothermia, whereas sham-lesioned rats successfully defended their *T*_b_ (Fig. 2*A*). However, when allowed to select their preferred *T*_a_ in a thermogradient apparatus, DMH-lesioned rats responded to a high dose of LPS with a smaller hypothermic response than sham-lesioned rats (Fig. 3*A*). How can the same lesion both promote and attenuate hypothermia? We show that these two effects differ in their thermoeffector mechanisms and DMH representations.

### Permissive effect of lesions on cold-induced hypothermia involves autonomic thermoeffectors only and is mediated by the DA

Both in our previous (Almeida et al., 2006b) and current (Fig. 2*A*) works, the *T*_b_ response to cold was studied in restrained rats that could not use behavior to select their preferred *T*_a_. The development of hypothermia in this setup was an autonomic (physiological) effect. A priori, it could involve impairment in any of the three thermoeffectors: nonshivering thermogenesis (in BAT and other tissues), shivering, and skin vasoconstriction in the specialized heat-exchange organs (which, in the rat, is primarily the tail). However, cutaneous vasoconstriction in the rat tail does not critically depend on the DMH (Rathner et al., 2008). In contrast, both BAT thermogenesis (the most important heat production effector in the rat; see Cannon and Nedergaard, 2004) and shivering critically depend on the DMH (for review, see DiMicco and Zaretsky, 2007; Morrison, 2016). For both effectors, glutamatergic DMH neurons receive inhibitory input from GABA-ergic preoptic neurons and project to glutamatergic premotor neurons in the rostral medullary raphe. In anesthetized rats, inhibition of DMH neurons with muscimol, a selective agonist of the GABA_A_ receptors, attenuates both BAT activation (Zaretskaia et al., 2003; Madden and Morrison, 2004; Nakamura and Morrison, 2007) and shivering (Tanaka et al., 2001; Nakamura and Morrison, 2011) induced by either skin cooling or the intrabrain administration of prostaglandin E_2_ (PGE_2_), the ultimate mediator of fever (Ivanov and Romanovsky, 2004). Lesioning the DMH electrolytically, thermally, or chemically renders unanesthetized rats incapable of defending their *T*_b_ autonomically against cold (Almeida et al., 2006b; present work).

Experiments in anesthetized rats with intrapreoptic administration of PGE_2_ have suggested that febrile thermogenesis is driven by the DMH (for review, see DiMicco and Zaretsky, 2007; Nakamura, 2015). However, the intrabrain PGE_2_ does not replicate the entire febrile cascade that occurs when an animal is challenged with an exogenous pyrogen (e.g., LPS) and anesthesia strongly affects the thresholds for cold defenses (Sessler, 2016). The present study is a first demonstration of the inability of unanesthetized DMH-lesioned rats to mount LPS fever autonomically (Fig. 4*B*). We also show that stress hyperthermia (associated with the brief restraint needed for drug administration in experiments in the thermogradient apparatus) is attenuated in DMH-lesioned rats (Fig. 3*A*,*B*). The latter finding agrees with studies in rats showing that disinhibition of DMH neurons by the GABA_A_ receptor antagonist bicuculline causes “stress-like” increases in BAT thermogenesis and *T*_b_ (Zaretskaia et al., 2002; Cao et al., 2004; de Menezes et al., 2006), whereas inactivation of DMH neurons by muscimol blocks stress hyperthermia (Kataoka et al., 2014).

Maps for the autonomic cold defense in unanesthetized rats show the strongest representation of this function in a site centered on the DA (Fig. 8), which agrees with multiple reports in anesthetized rats cited above. The location and size of our site correspond well to the area of the highest density of DMH neurons retrogradely labeled from the raphe (Samuels et al., 2004; Nakamura et al., 2005; Yoshida et al., 2009; Kataoka et al., 2014), especially of those subsets of the raphe-projecting neurons that express c-Fos in response to cold (Cano et al., 2003; Yoshida et al., 2009) or social stress (Kataoka et al., 2014). Our site also partially overlaps with the DMH areas in which neuronal activation produces tachycardia (Samuels et al., 2004; Tanaka and McAllen, 2008) and a plantar vasoconstrictor response (Tanaka and McAllen, 2008), but does not overlap with the area triggering the phrenic nerve response (Tanaka and McAllen, 2008).

### DMH lesions attenuate hypothermia in LPS shock by blocking cold-seeking behavior, an effect mediated by neurons at the DM–VMH border

In our earlier (Almeida et al., 2006b) and present (Fig. 3*A*) studies, a blockade of cold-seeking behavior clearly contributed to the attenuation of LPS-induced hypothermia in DMH-lesioned rats, but this does not exclude an involvement of autonomic mechanisms. When we measured the autonomic thermoeffectors, we found that DMH-lesioned rats never responded to LPS with higher thermogenesis or stronger tail-skin vasoconstriction than sham-lesioned rats (Fig. 4). Therefore, the attenuation of LPS-induced hypothermia by DMH lesions (Fig. 3*A*) is solely due to an effect on cold-seeking behavior.

Functional maps show that the site for this behavior is located, not in the DA, but at the border between the middle aspect of the DM and the caudal aspect of the VMH (Fig. 8). When ibotenic acid was injected into this site to lesion neuronal bodies (while sparing fibers of passage), rats with such injections did not seek cold in response to LPS, but had an intact autonomic defense against cold (Fig. 10). Therefore, LPS-induced cold-seeking behavior critically depends on neuronal bodies at the newly identified DM site, but not on the DA. Our results to some extent mirror the reports that implicated DM (but not DA) neurons in the circadian control of multiple behaviors (Chou et al., 2003; Gooley et al., 2006).

LPS-induced cold seeking also critically depends on neural fibers passing through the PVH, because large electrolytic lesions, but not excitotoxic lesions, of the PVH block this behavior (Almeida et al., 2006b). Because rats with electrolytic lesions of the DMH or PVH have normal behavioral thermoregulatory responses to ambient cooling and warming (Almeida et al., 2006b), the DMH and PVH are involved in a stimulus-specific (LPS-induced) component of cold seeking. We speculate that this could be the affective or motivational (but not sensory or motor) component. The DMH sends marked projections to the septum and amygdala (ter Horst and Luiten, 1986; Thompson et al., 1996; Onat et al., 2002), the structures that control the affective and motivational components of various behaviors (Gallagher and Chiba, 1996; Phelps and LeDoux, 2005; Heimer and Van Hoesen, 2006). Moreover, the septum has been shown to drive warmth-avoiding (cold-seeking) behavior in rats (Roberts and Mooney, 1974). A major route for ascending projections from the DMH runs through the periventricular zone, including the Pe (Thompson et al., 1996; Thompson and Swanson, 1998), and this route is likely to be interrupted by electrocoagulation of the PVH, but not by lesioning with ibotenic acid. The site revealed in the present study may be overlapping with the areas that contain PVH-projecting neurons activated by leptin (Elmquist et al., 1998) or stress (Kataoka et al., 2014).

Our DM site is also anatomically similar to the area containing neurons that express neuropeptide FF (NPFF), which is thought to mediate analgesia and modulate spatial recognition and arterial blood pressure (Panula et al., 1999; Ayachi and Simonin, 2014). According to Aarnisalo and Panula (1995), this area is “located in the medial hypothalamus between the dorsomedial, ventromedial, and periventricular hypothalamic nuclei” and has strong projections to the limbic system and the Pe. The limbic targets of NPFF-containing neurons are the septum, BNST, and several amygdalar subnuclei. The septum and BNST also have some of the highest densities of type-1 NPFF receptors in the rat and human brains (Bonini et al., 2000; Goncharuk et al., 2004).

Cold-seeking behavior in systemic inflammation may be considered a transition symptom between the motor agitation, hyperalgesia, hypertension, and fight/flight behavior characteristic of mild systemic inflammation and the motor depression, hypothermia, analgesia, hypotension, and behavioral withdrawal characteristic of severe inflammation (see Introduction). This transformation of the sickness syndrome, occurring when mild inflammation becomes severe, resembles the progression of symptoms during innate fear responses to environmental threats. In these responses, escape behavior, tachycardia, hypertension, and other dramatic manifestations of panic are followed by a depression/withdrawal state and antinociception (Freitas et al., 2013). Remarkably, this behavioral sequence can be triggered by disinhibiting neuronal clusters in the ventral DM or dorsomedial DM (VMHDM) with bicuculline (Freitas et al., 2013; Dos Anjos-Garcia et al., 2017). Furthermore, both the panic behavior and subsequent analgesia can be attenuated by a cannabinoid-1 (CB1) receptor antagonist administered in the VMHDM (Dos Anjos-Garcia et al., 2017) or prelimbic medial prefrontal cortex (Freitas et al., 2013). Both structures contain CB1 receptors (Wittmann et al., 2007; Tan et al., 2010; Reguero et al., 2011). Intrabrain (but not peripheral) CB1 receptors have also been implicated in the development of LPS-induced hypothermia in rats (Steiner et al., 2011).

### Conclusions

By conducting experiments in conscious, unanesthetized rats and using a functional mapping technique, we show that the DMH contains at least two thermoregulation-related sites. The first site is centered in the DA and is critical for the autonomic defense of *T*_b_ against cold, as well as for mounting the fever response to LPS and stress hyperthermia. The second site is located at the ventral border of the DM. Neurons at this site are critical for LPS-induced cold-seeking behavior, perhaps the affective or motivational component of this behavior.
